# Forecast or Fall: Prediction's Importance to Postural Control

**DOI:** 10.3389/fneur.2018.00924

**Published:** 2018-10-30

**Authors:** Christopher J. Dakin, David A. E. Bolton

**Affiliations:** Department of Kinesiology and Health Science, Utah State University, Logan, UT, United States

**Keywords:** fall, balance, posture, prediction, anticipation, postural control

## Abstract

To interact successfully with an uncertain environment, organisms must be able to respond to both unanticipated and anticipated events. For unanticipated events, organisms have evolved stereotyped motor behaviors mapped to the statistical regularities of the environment, which can be trigged by specific sensory stimuli. These “reflexive” responses are more or less hardwired to prevent falls and represent, maybe, the best available solution to maintaining posture given limited available time and information. With the gift of foresight, however, motor behaviors can be tuned or prepared in advance, improving the ability of the organism to compensate for, and interact with, the changing environment. Indeed, foresight's improvement of our interactive capacity occurs through several means, such as better action selection, processing, and conduction delay compensation and by providing a prediction with which to compare our actual behaviors to, thereby facilitating error identification and learning. Here we review the various roles foresight (prediction) plays in maintaining our postural equilibrium. We start by describing some of the more recent findings related to the prediction of instability. Specifically, we cover recent advancements in the understanding of anticipatory postural behaviors that are used broadly to stabilize volitional movement and compensate for impending postural disturbances. We also describe anticipatory changes in the state, or set, of the nervous system that may facilitate anticipatory behaviors. From changes in central set, we briefly discuss prediction of postural instability online before moving into a discussion of how predictive mechanisms, such as internal models, permit us to tune, perhaps our highest level predictive behaviors, namely the priming associated with motor affordances. Lastly, we explore methods best suited to expose the contribution of prediction to postural equilibrium control across a variety of contexts.

## Introduction

**Figure 1:**
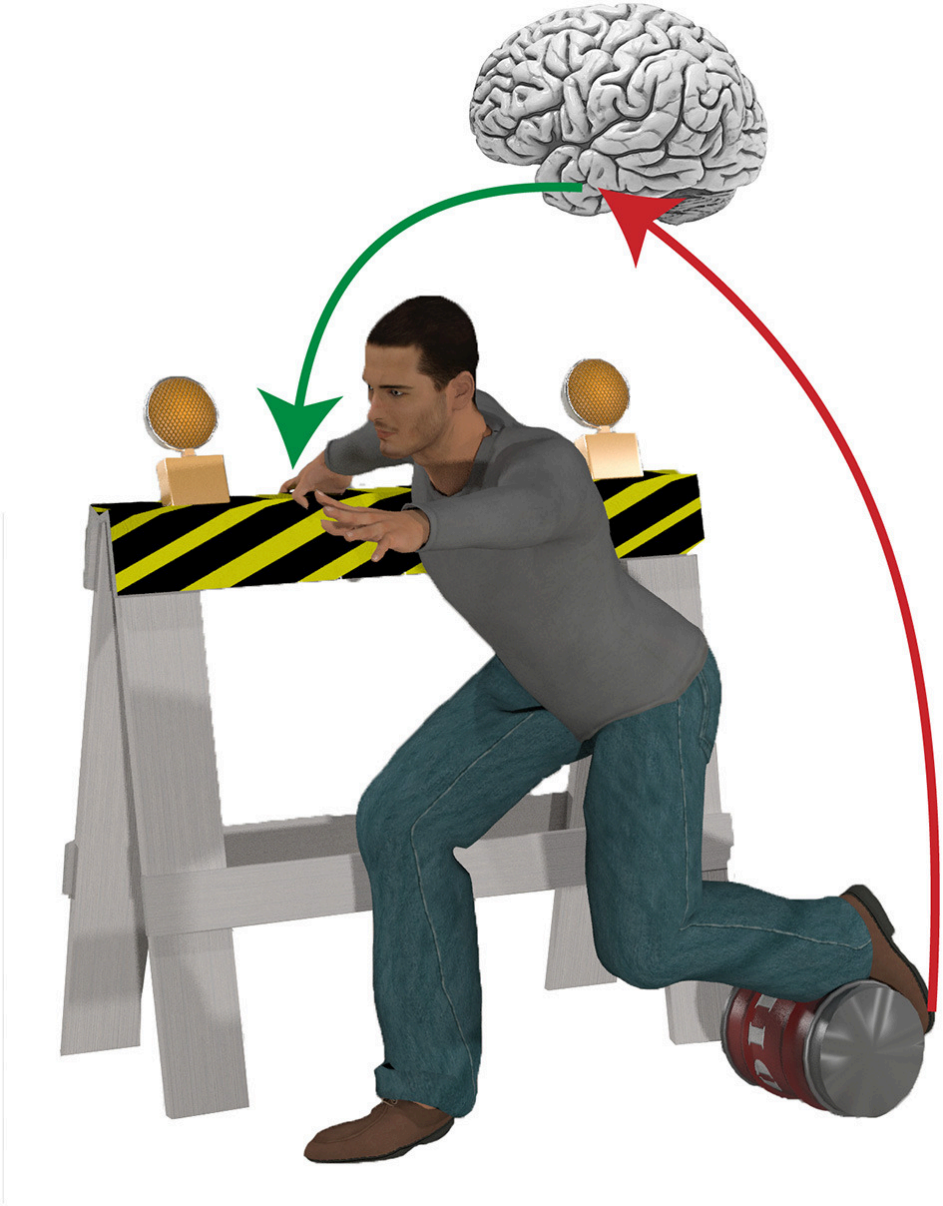
Dealing with complex environments often requires behavioral flexibility to maintain postural equilibrium. For example, in cluttered environments it is often necessary to grasp a nearby object to establish a new base of support, while suppressing a highly automatic stepping reaction if an obstacle blocks the foot. The speed and complexity of such sophisticated, goal-directed behaviors necessitates a higher level of control, and implicates a role for advanced preparation based on environmental cues in the control of balance.

The world is full of obstacles, opportunities and distractions with which we must interact. Some of these interactions are simple, permitting a reliable stereotyped response with each occurrence, while others are more complicated, requiring more refined pattern recognition, and decision making mechanisms. All of these interactions operate under the constraint of time. Traditionally, balance (the act of maintaining postural equilibrium) studies have favored simple (i.e., unobstructed) environments, where cues can be controlled, and where response settings are purposely unadorned in an attempt to isolate putatively pure elements of balance control. In many of these types of study, the central nervous system can maintain postural equilibrium using relatively simple righting mechanisms embedded within the most basic levels of the neural hierarchy (e.g., spinal cord and brainstem) thereby minimizing processing delays. However, many real-life falls occur in complex environments that require flexible decision-making mechanisms (Figure 1). Here, a much more distributed neural network must play a role, but this comes at a cost. Processing information in a more expansive network can render a “better” decision but one that is too late to be effective. The solution to this problem is to predict and prepare for our future interactions.

Accumulating evidence indicates that a broad network of high-level neural structures with known adaptive and predictive functions, including the cerebellum, basal ganglia and cerebral cortex, contribute to maintaining postural equilibrium. Given the capacity of these “cognitive” networks to process current and historical information, they are ideally suited to recognize current context in the light of previous experience for the purpose of dynamically anticipating and preparing for action. Such flexibility is important as we move through the world because the actions that are ideal for maintaining postural equilibrium will change with the constraints and opportunities afforded by a particular environment. The additional need to select the most appropriate response from an array of options, while simultaneously suppressing unsuitable, yet potentially automated actions, implies a need for higher-level supervision. It also raises the question of how we combine the utility of rapid, stereotyped compensatory reactions with the need to match our actions to what is permitted by a given environment at a particular moment in time? Insight into this question may arrive from fields of study not traditionally associated with postural control, such as cognitive psychology and even artificial intelligence. This cross pollination of ideas across multiple fields broadens how we view the neural control of balance (1, 2). Specific cross-discipline concepts such as predictive modeling (internals models), affordances for action, and associative learning each have important implications for adapting our movements to maintain postural equilibrium in challenging environments, and provide a way to overcome conflicting demands for goal-directed action at high speed.

### Qualifying statements

Before we begin to examine these concepts, we would like to make clear that the aim of this review is to highlight the many means by which prediction can contribute to postural, and by extension, movement control. Additional compensatory mechanisms are also crucial to the maintenance of our stability, and their omission here is not meant to minimize their importance, but to highlight the oft-neglected contribution prediction plays in these behaviors. In addition, throughout this exploration of prediction we have avoided categorizing predictions based on whether it contributes to a volitionally driven events or to the compensation of externally induced events. Instead, we would like the reader to focus on the importance of certainty in a prediction's utility.

## Predicting instability

### Anticipatory postural adjustments

To prevent destabilization of the body during volitional movement, postural changes meant to compensate for the disturbance generated by the movement, precede the movement itself (3–7). These anticipatory postural adjustments, or APAs, represent the culmination of a predictive process that estimates the postural disturbance associated with an impending movement. In order to support effective movement APAs must be highly adaptable to enable the close correspondence required between the voluntary act and its associated stabilizing activity.

The generation of APA's, and movement, involve broad interconnected networks that span much of the central nervous system: from the lowest levels of the spinal cord to the brainstem, and ultimately the cerebral cortex. These expansive networks provide the computational power necessary to flexibly adapt APAs to complex and uncertain environments. The spinal circuitry, for example, can be set in advance to shape imminent APA's (see the next section) while further up the neural hierarchy, the brainstem contributes to the coordination and, perhaps, generation of APA's and subsequent movement. Recording electrodes within the pontomedullary reticular formation in the brainstem of cats reveal a population of neurons that operate as a coordinated unit to control one forelimb during a voluntary reach while stabilizing posture with the other forelimb (8). This observation suggests a basic substrate for linking body segments to provide stability suited to movement demands. In addition, within this region there are also separate populations of neurons that encode the initiation of APAs, the initiation of volitional movement and the combination of the two (9). The divergence in the encoding of APA's and movement suggests at least some independence of the mechanisms underlying the two of them but regions that generate these predictions remain unclear. As we will see, independent encoding of the APA and movement is also prevalent further up the neural hierarchy.

Several higher-level neural networks including the basal ganglia, cerebellum and cerebral cortex—all with roles in learning and adaptation (10)—have also recently been associated with the generation and implementation of anticipatory postural behavior and therefore prediction (11–19). The basal ganglia, for example, is proposed to facilitate reinforcement learning (10) and coincidentally patients with Parkinson's disease, are less able to adapt their APAs to novel contexts (20), in addition to having smaller amplitude and even delayed responses in some instances (21–23). Similarly, the cerebellum is associated with error-based supervised learning, which contributes to the adaptability of APAs. This adaptability is impaired in patients with cerebellar degeneration (15) and such disorders can also lead to changes in the shape and/or timing of APAs (12), but this latter point remains a topic of debate as there are some reports that well-learned relationships remain largely intact following degeneration (24). Lastly, the integrative processing power of the cerebral cortex also provides the functionality to develop high-level associations between sensory stimuli and context specific responses. Recent evidence suggests that both the supplementary and primary motor cortices contribute to the generation of APAs. The supplementary motor area modulates the size of the APAs independently of the associated volitional movement, implying that the APA and movement are represented separately at this stage of processing (11). In addition, patients with lesions of the supplementary motor area have impairments in the shape and timing of their APAs (25) which can be loosely simulated in healthy adults by functional lesion of the supplementary motor area using repetitive transcranial magnetic stimulation (18). In contrast, the primary motor cortex maintains a shared representation of both APA and movement and is proposed to shape the amplitude and timing of APAs (13, 17, 25–28). In general, the cerebral cortex appears to play a key role in refining the mapping of postural adjustments to voluntary acts. As discussed next, cortical involvement in predictive postural control extends beyond the APAs that precede self-initiated movement. The observed increase in excitability of projections from the primary motor cortex to the spinal cord that occurs prior movement (14, 17) implies that the cerebral cortex contributes to setting the state of the spinal cord in anticipation of a postural disturbance so that the spinal circuitry behaves appropriately in the event of a disturbance.

### Central set

Explicit awareness of a forthcoming perturbation is perhaps the most obvious scenario where one can envision a prominent role for prediction in compensatory balance. Essentially many features of a response can be covertly prepared in advance by setting the state, or set, of the central nervous system via descending commands, thereby reducing delays associated with stabilizing an impending movement or generating an appropriate counter reaction. In a seminal exploration of central set, Horak et al. used a fixed-support, platform translation paradigm to investigate systematically the relative influence of central set vs. peripheral drive on generating automatic postural responses (29). Their study exposed participants to varying magnitudes of postural perturbation while researchers controlled the amount of information provided in advance about the size and speed of the impending perturbation. They found that participants scaled the amplitude of their early muscle responses to the expected amplitude of the perturbation, particularly after repeated exposure to a specific platform translation. This result demonstrated that the central nervous system shapes the amplitude of muscle responses based on a prediction, developed over time, of what is going to happen. Presumably, such advance preparation reduces or eliminates the delay with which the body can respond to a perturbation and helps shape the body's response to the perturbation (30–32).

Since the seminal findings of Horak et al. (29), researchers have used more direct measures of corticospinal excitability and spinal reflex modulation to reveal the preparatory activity that occurs in spinal and cortical networks in advance of a predictable perturbation (33). Several electroencephalography studies have shown that prior to a predictable postural disturbance, a slow wave potential builds under central scalp electrodes (34–36). This potential continues to build until the postural disturbance occurs, at which point a separate post-perturbation cortical potential known as the N1 response is observed (36). More recent studies have shown that this anticipatory cortical activity is similar regardless of whether the disturbance is self or externally induced (37) and it scales with the amplitude of the impending perturbation (32). Concurrent changes in the circuitry of the spinal cord accompany these anticipatory cortical potentials (33) implying that the purpose of this cortical activity may be to modify the “central set” or state of the nervous system (35), however, a causal relationship between the two remains to be defined.

### Dynamic prediction of instability and sway

Sensory signals indicating an impending loss of balance can stimulate preparatory changes throughout the nervous system to compensate for the future disturbance to equilibrium. Such advance signaling is important because without prediction, online estimates of body position rely on outdated information due to the lag in signal transmission. In many large postural disruptions, advance preparation is necessary to maintain postural equilibrium because there is insufficient time to respond to the disturbance. Thus, the central nervous system must consistently monitor sensory information for evidence of a threat to stability in order to recognize events in advance that might require postural compensation. In some instances, such as during standing balance, specific characteristics of the sway pattern can provide predictive cues as to whether intervention is necessary to maintain postural equilibrium. Virtual time to contact (VTC) has been proposed as a low dimensional variable that the central nervous system could monitor in order to predict instability during standing balance (38). Specifically, VTC is defined as the time it would take for the body to reach the boundary of our stability if it were to continue on its current trajectory from its current state (position and velocity) with constant acceleration (39). When nearing a loss of balance, changes in the minimum value and variance of the VTC measure are correlated with changes in electroencephalographic power estimated at the anterior cingulate cortex, precuneus and the parietal and occipital lobes (40). The authors propose that since these concomitant changes in VTC and accompanying neural markers precede loss of balance, they provide a predictive cue to the future instability of our posture.

Certain populations may also be at increased risk of falling because they fail to use the most recently available data to predict a coming disturbance to balance. For example, when stepping is induced by an external perturbation, older adults (especially those with higher fall risk) step earlier than young adults once they detect a postural threat, even though the perturbation could have been managed by using a fixed-support reaction (41). This earlier step appears to be a default strategy, absent appropriate scaling to the disturbance, and one that is often insufficient to compensate for the disturbance, thus requiring multiple follow-up adjustments/steps. These findings underscore the value of accurately interpreting the evolving sensory state in the brief time prior to the fall to the generation of appropriate and appropriately scaled corrective actions.

Postural sway itself is also often thought to be, at least partially, the product of a predictive control mechanism. Fitzpatrick et al. (42) examined the gain of postural reflexes during human standing and found it insufficient to maintain postural equilibrium on its own (42). Because of this insufficiency, the authors concluded that the control of sway must involve a feed-forward control component. Moreover, a positive phase shift in lateral gastrocnemius muscle activity relative to ankle loading has also been observed during maintenance of postural equilibrium (43). If sway used only sensory feedback to control ankle muscle stiffness, the muscle's activation pattern should lag ankle loading. In reality however, muscle activation in the lateral gastrocnemius precedes ankle loading suggesting the timing of muscle activation likely involves an anticipatory process. Gatev et al. (43) also questioned whether the sway observed during postural equilibrium is secondary to the control process, i.e. random variance associated with maintaining postural equilibrium, or whether it is the intended consequence of an exploratory control process (43). Recently this hypothesis has been tested and has seen further support (44, 45). Under this control scheme, sway is potentially promoted to allow exploration of the base of support. If true, such exploration could involve the use of forward models (discussed in more depth below) to predict the sensory consequences of the exploration in order to isolate better deviations from expectation. Support for a predictive contribution to sway also arises from the unique control scheme required to control the lower limb during standing balance. Due to a poor match between the stiffness of the connective tissues and musculo-tendinous unit at the ankle, and the load-stiffness of the body (46, 47), the central nervous system is thought to activate the muscles of the lower limb in a predictive and intermittent manner (48–52). However, a recent modeling effort suggests that some of these behaviors could also emerge without a predictive control mechanism (53).

### Predicting the consequences of ones actions–internal models

One of many important functions of the central nervous system is to learn relationships. These relationships represent our understanding of how our body interacts with itself and the world. In motor control, one prominent encapsulation of these relationships is the abstract concept of an internal model. Internal models represent a learned relationship that can be used, among other things, to generate or simulate behavior, and conceptually it may provide a useful framework to understand how the brain might develop contextually appropriate compensatory behaviors. Neural networks that map a movement to its outcome are called forward models, and they can be used to simulate the sensory consequences of one's actions. Because of their ability to predict movement outcomes, forward models are believed to serve an important role in the supervised learning mechanisms associated with the cerebellum (54–56) by permitting comparison between what the body expects to sense as a consequence of movement, to what it actually senses. Differences between the expected and actual sensory feedback can represent a stimulus that requires compensation, or a prediction/movement error that requires adaptation. A forward model could also be useful to calculate the postural compensation necessary for anticipated disturbances to postural equilibrium. Such mechanisms are proposed to contribute to the control of precision grip because grip force leads changes in grip load. This anticipatory gripping behavior is thought to arise because the central nervous system maintains a model of limb and load dynamics that it uses to generate predictions of the load force acting on the hand, in order to preemptively compensate for anticipated changes in load (57, 58). The use of a forward model is also thought to contribute to stability during proactive stepping and obstacle clearance. Specifically, when taking a step, the body's weight is first shifted to one leg to maintain stability while the other leg is lifted. In order to transfer weight effectively the body may use an internal forward model to estimate whether the APA's have achieved a sufficient shift of the body's weight to maintain stability while the other leg is lifted (41). A similar mechanism aids stability during obstacle avoidance while stepping. In this context, the CNS is thought to predict the destabilizing effect of gravity acting on the body to allow the development of APA's appropriate for controlling body posture while in a single leg stance (59).

In each of the above examples, the forward model provides predictions that are used in the generation of contextually appropriate behavior. However, the behaviors generated from the forward model's predictions could also be formalized as a second type of internal model known as an inverse model. Inverse models receive sensory predictions and generate the motor commands necessary to create the sensory prediction. When paired with a forward model the combined neural network constitutes a means to generate motor compensation for predicted states of the body. Such a network has been proposed as a model for general sensorimotor learning and control (60–62) and is equally applicable to the subfield of postural control. Indeed, paired forward-inverse models could perform the duties of predicting the postural disturbance as well as generate the appropriate compensatory response from the prediction. From a general perspective, the power of internal models has already been suggested to have evolved in order to contend with conduction velocity delays with a larger body size (63), and this seems particularly relevant in the time-pressured world of compensatory balance.

## Pattern recognition and learned associations guide future action

Some patterns occur with such frequency that behaviors are seemingly hard wired into the nervous system. For example, coupling of the motion between body segments occurs so frequently that the movement of one limb often modifies the behavior of another. Modulation in the excitability of motor neurons in the upper limbs often occurs during lower limb tasks, and it suggests an anticipated cooperative integration of the extremities. Normally this contextual modification of motor neuron excitability occurs covertly, but if the limb becomes engaged in a postural task, these changes in excitability can become overt, resembling anticipatory postural responses (64–66). The behavior that results from such anticipatory or linked activities depends on the mechanical or sensory context under which they arise. For example, Esposti and Baldissera (67) suggest that there is an arborized pattern of behaviors from which one, or a select group, could become released from inhibition to affect behavior depending on the context. Release of such behaviors could arise via anticipatory mechanisms or a change in the mechanical or sensory context, such as the innocuous expectancy of visual information (68). These types of behaviors likely also contribute to the higher-level associations that allow us to effectively navigate and interact with complex environments.

The surrounding environment in which we generate a compensatory response is often filled with obstacles and distractions competing for our attention. The increased attentional resources and behavioral flexibility required to navigate these environments presumably raises the risk of a fall [For a comprehensive account of typical causes for falls in an assisted living setting and their relative incidence, see (69)]. As the complexity of the environment increases, the ability to recognize environmental patterns that support successful goal-directed action (and cueing on the most relevant pieces of the scene) becomes more important. For example, during an athletic competition, preparing a menu of possible behaviors based on fragmented, preliminary data can increase efficiency when performing under the time pressure of sports (70). Furthermore, experts are better at identifying relevant information from the visual scene, and do it much quicker than novices, indicating such pattern recognition can be learned. While this example pertains to performance in sport, it is also relevant to postural stability. Specifically, predictive cueing offers a way that the brain can use environmental stimuli presented at a much earlier point in time to identify a potentially successful behavior in the event that it is needed in the future. Central to the concept of predictive cueing is the idea that successful interaction with a specific object is strongly associated with a particular action. For example, a mug with handle is associated with a grasp, whereas while walking, an uncluttered space on the ground in front of you is likely the best location for the next step. Learned associations between the object and our actions such as these are reliant on experience interacting with the world in a variety of contexts. Moreover, the development of these associations likely depends upon the learning and associative power of cortical, basal ganglia, and cerebellar networks as mentioned earlier (10).

### Affordances for action and the relevance to balance recovery

**Figure 2:**
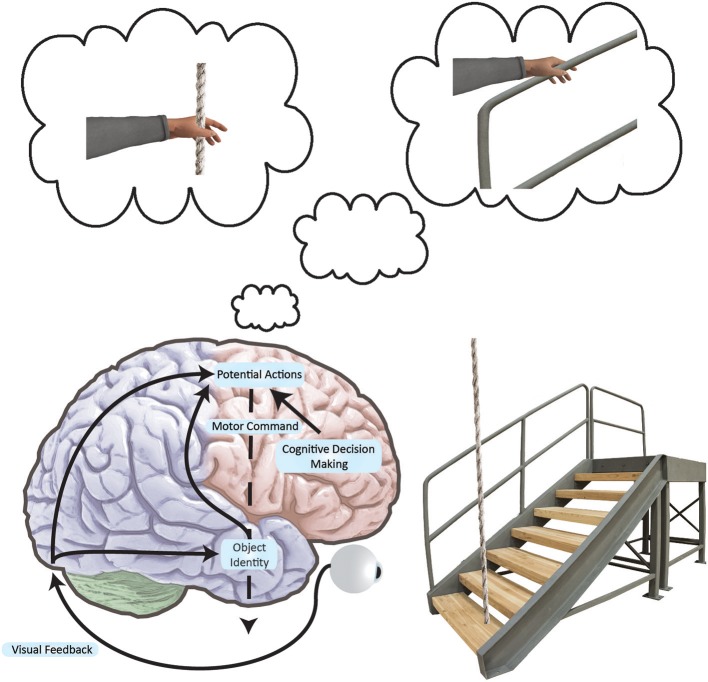
Simplified neural networks underlying an affordance to grasp. Black arrows indicate how the brain converts visual information into movement plans for a variety of possible actions. As movements are encoded in frontal and parietal networks, action representations compete with one another. These actions are biased by the basal ganglia and prefrontal cortex at multiple locations in the brain as per the affordance competition model (103, 104). We act when one of the possible actions wins the competition. In this example, we see that the stairs have a supporting railing. The railing affords a grasp and the rope affords a grasp, but since the railing is more stable, the railing grasp is primed. If the grasp is the most salient afforded action, we may execute it in the event of a stumble. Here, it is important to note that such directed arm action would conceivably be prompted by viewing a supportive handle—a handle associated with postural recovery from past experience. Furthermore, such action would only exist as an internal representation until called upon.

Considerable evidence from animal (71–74) and human research (75–82) has shown that viewing objects strongly associated with particular actions can potentiate these actions, suggesting that we encode our surroundings in terms of the movements the surroundings afford (Figure 2). This concept, known as “affordances” (83), has been demonstrated in humans using various neuroimaging and stimulation techniques, including functional Magnetic Resonance Imaging (78, 79) and Transcranial Magnetic Stimulation (TMS) (75–77, 80, 84), as well as behavioral outcomes such as improved reaction time (81). The predictive nature of visual priming based on these affordances is especially relevant given the processing delays inherent to a large, complex nervous system. Such a predictive mechanism potentially holds great value for controlling postural equilibrium.

Although control of postural equilibrium was long thought to be mediated subcortically (85, 86), a large body of evidence now attests to the involvement of the cerebral cortex in the control of postural equilibrium, including compensatory reactions to unexpected postural challenge (32, 33, 87, 88). Perhaps most crucial are compensatory reactions that require the limbs to establish a new base of support and catch a falling center of mass (89–91). Notably, these change-of-support reactions are the only line of defense when a disturbance to posture exceeds a certain threshold. The fact that high-level neural networks can play a role in responding to unexpected external postural perturbations seems remarkable given how quickly these whole-body responses must take place to avoid a fall. However, if suitable responses could be established *prior* to a fall, this would offer a viable solution for producing fast, yet sophisticated “context-appropriate” reactions. Thus, motor affordances could potentially bias specific recovery actions suited to our surroundings, even before the need for such action.

Since Gibson first presented the concept of affordances many years ago (83) several lines of evidence support the basic idea of affordances including animal studies that have identified premotor neurons activated by the mere appearance of graspable objects known as *canonical* neurons (74). Furthermore, human studies have demonstrated a measurable link between simple object observation and motor cortical activation even with no requirement to move (77). Remarkably, the rare clinical condition of alien hand syndrome (which sometimes results from a stroke in the frontal lobes) also offers support for this idea (92). These patients lack inhibitory oversight and instead are irresistibly compelled to interact with surrounding objects. These interactions are not random but give the appearance of goal-directed movement, despite a reported lack of intention to move.

While these concepts have not been considered in the domain of compensatory balance reactions, the potential applicability of setting contingent responses based upon the environment in advance is clear. Consider for example walking down a hallway with a handrail anchored along the wall. According to the notion of perceived affordances, arm movements may be prompted by simply viewing these handrails while any overt movement would remain dormant (or actively inhibited) until needed. In this instance, one can begin to see how such a mechanism holds great relevance for enlisting a rapid reach-to-handle reaction if a challenge to postural equilibrium occurs. Essentially, a contingent motor response may be automatically dictated by perception of the surrounding world and called into action (or released from inhibition) when circumstances warrant this action. Recently, the excitability of corticospinal projections to specific grasping muscles was shown to be modulated when participant's simply viewed a wall-mounted safety handle (93). This result provides some initial support for the idea that viewing an object associated with balance recovery can modify central nervous system activity.

Another important consideration when encoding the world in terms of afforded motor actions is that sometimes the environment will contain obstructions to potential actions. Understandably, the central nervous system should avoid priming actions that bring the body into an obstruction, requiring the inhibition of inappropriate actions. Inhibition is particularly important in situations when postural equilibrium is disturbed and there is an obstacle preventing a recovery step. In such a case, equilibrium would normally be recovered by taking a forward step to prevent a forward fall, but doing so would accentuate the fall. This stepping response is salient given the highly automated nature of a recovery step used to recapture a falling center of mass (90, 91). Thus, an important aspect of pre-setting compensatory behavior prior to a fall would involve facilitation of appropriate action, as well as suppression of pre-potent but inappropriate action based on environmental context. The ability to override automatic, but unwanted actions and to filter out distracting information, ultimately relies upon oversight by the prefrontal cortex (94) suggesting it may play an important role in fall resistance.

## Neural networks involved in planning future actions

At this point, we have reviewed various predictive mechanisms that could contribute to the control of postural equilibrium. Essential to all these mechanisms is the capacity to adapt or learn from experience to inform future action. Not surprisingly, a commonality among the various aspects of prediction is their association with cortical, basal ganglia and cerebellar networks. Each of these anatomical regions has been proposed to implement its own unique learning algorithm that could be used to develop and refine posture related predictions (10). Learning in the cerebral cortex is thought to occur through Hebbian plasticity, an “unsupervised” learning mechanism. Hebbian plasticity is based on the idea that temporally synchronous and causally related firing among networks of neurons results in a strengthening of the relationship between the two networks. This form of learning attempts to “map” associations in which a quantifiable error signal is absent and this 'mapping' may underlie the recognition and association of sensory cues deleterious to posture with their appropriate response. The basal ganglia, in contrast, is thought to shape our behavior through reinforcement learning. Reinforcement learning is a process where correct behaviors are rewarded to facilitate learning and this reward signal, and subsequent change in reward signal, is encoded by dopaminergic fibers from the substantia nigra within the basal ganglia (10). Behaviors, such as motor action, are selected in this learning paradigm by maximizing the predicted reward that each option could bring. Such a mechanism could be ideal for the selection and reinforcement of appropriate compensatory actions resulting from a loss of stability. Lastly, the cerebellum is proposed to implement an error based “supervised” learning mechanism whereby the consequences of our actions are predicted and compared to reality. The difference between the prediction and reality can be used to adjust our predictions, but also represents a disturbance to posture that must be reactively compensated for. Together, with the thalamus, these neural networks develop the associations between particular contexts, probable scenario's and appropriately matched compensatory behaviors that sub-serve predictive postural control as well as refine reactive postural mechanisms. As a final point, a characteristic of predictive control is the ability to regulate relative timing of events, such as the coupling of an APA prior to stepping. For example, a voluntary step would need to be actively delayed until sufficient weight transfer occurs through an APA (41). Such control over relative sequencing of events relies upon a time buffer or memory of sequence fragments (95), which suggests cerebellar involvement (96), as well as prefrontal cortex due to its important role in working memory (97, 98).

## Improving experimental design to expose and emphasize predictive roles

A big reason we fail to understand prediction's contribution to reactive balance may be due to the simplicity of research protocols that are frequently used. The status quo in postural equilibrium research is to provide relatively small perturbations in clutter-free environments, with an emphasis on fixed support (feet-in-place) reactions. However, when perturbations are large, change-of-support reactions are the only option to recovery stability (90, 91). Daily life often imposes obstacles, while also providing various movement options that can help us regain balance. In some cases, obstacles force a selection process requiring a limb to target a new support base if a loss of balance occurs. As the need for behavioral adaptation rises, so does the demand on higher brain resources (and foresight), particularly when we use the arms or legs to establish a new base of support amid complex surroundings. To truly emphasize the contribution of prediction to reactive balance, researchers may need to reintroduce the clutter and force a change-of-support strategy with the limbs.

Reliance on external measures such as muscle onsets, ground reaction forces, and video motion capture to infer neural processes may also limit our perspective on the control process involved in maintaining posture. Such external measures can miss what the central nervous system is doing to help us avoid a fall. In fact, this problem is compounded when you consider that much of what the brain may do to prevent a fall in complex environments may happen *before* the fall. This includes predicting future instability (40), building visuospatial maps as we move through our environment (99), and possibly forming contingencies based upon the environment even without foreknowledge of a fall (100). Without the use of direct neurophysiological probes, it is difficult to reveal such preparatory behavior.

Study designs that emphasize direct neural measures and change-of-support reactions within cluttered environments pose significant methodological challenges. However, these study designs also have great potential to reveal the predictive mechanisms underlying fall avoidance in the complex settings encountered in daily life. Thus, using direct neurophysiological stimuli or measures such as Transcranial Magnetic Stimulation (TMS), Electroencephalography, and/or functional Near-infrared Spectroscopy in the period *before* and after postural perturbation could provide important experimental advances. Furthermore, research designs where the limbs are required to establish a new base of support, all within cluttered and choice-demanding environments, could expose higher brain processes where prediction is necessary to respond appropriately to a loss of stability. This combination of experimental features represents an important innovation in the field to expose how the brain contributes to fall resistance in complex, real-life settings.

## Conclusions

Some of the ways in which prediction contributes to balance are now well understood, particularly in cases where self-initiated movement needs to be stabilized or when we need to counter a known perturbation originating from an external source. Both of these instances hinge on past-experience and learning to match postural adjustments with an internal representation of the forthcoming disruption to equilibrium. Perhaps less intuitive is how predictive mechanisms can operate behind the scenes to prepare contingent actions based on cues and contexts that have been implicitly acquired through interactive experience with the world. Such associative learning has been studied in fields outside the domain of postural control, but may hold great significance for regulating postural equilibrium in an unstable and choice-demanding world. Prediction, in theory, offers an important way that higher neural networks contribute to speeded recovery actions. Indeed, the need to forecast future instability and plan appropriate countermeasures may explain (at least partly) the correlation between cognitive decline and falls (101, 102). Determining how predictive mechanisms impact balance recovery will require some revision to traditional research paradigms that “start the clock” only after the perturbation has occurred. Furthermore, the use of simplistic lab settings may fail to sufficiently expose a need for predictive mechanisms and inadvertently bias our understanding of how balance is controlled to favor lower reflexes. Research designs frequently operate from a framework where postural reactions are purely reactive without the help of foresight. Therefore, broadening this perspective to consider the potential role for prediction in the field of balance control could begin to fill an important gap in understanding the mechanisms for how cognitive resources influence resistance to falls.

## Author contributions

All authors listed have made a substantial, direct and intellectual contribution to the work, and approved it for publication.

### Conflict of interest statement

The authors declare that the research was conducted in the absence of any commercial or financial relationships that could be construed as a potential conflict of interest.
